# MEMS Devices-Based Hand Gesture Recognition via Wearable Computing

**DOI:** 10.3390/mi14050947

**Published:** 2023-04-27

**Authors:** Huihui Wang, Bo Ru, Xin Miao, Qin Gao, Masood Habib, Long Liu, Sen Qiu

**Affiliations:** 1 School of Intelligence and Electronic Engineering, Dalian Neusoft University of Information, Dalian 116023, China; wanghuihui@neusoft.edu.cn (H.W.);; 2 Key Laboratory of Intelligent Control and Optimization for Industrial Equipment of Ministry of Education, Dalian University of Technology, Dalian 116024, China; 3 College of Aeronautical Engineering, Taizhou University, Taizhou 318000, China

**Keywords:** inertial sensor, gesture recognition, support vector machine, hidden markov model, deep learning

## Abstract

Gesture recognition has found widespread applications in various fields, such as virtual reality, medical diagnosis, and robot interaction. The existing mainstream gesture-recognition methods are primarily divided into two categories: inertial-sensor-based and camera-vision-based methods. However, optical detection still has limitations such as reflection and occlusion. In this paper, we investigate static and dynamic gesture-recognition methods based on miniature inertial sensors. Hand-gesture data are obtained through a data glove and preprocessed using Butterworth low-pass filtering and normalization algorithms. Magnetometer correction is performed using ellipsoidal fitting methods. An auxiliary segmentation algorithm is employed to segment the gesture data, and a gesture dataset is constructed. For static gesture recognition, we focus on four machine learning algorithms, namely support vector machine (SVM), backpropagation neural network (BP), decision tree (DT), and random forest (RF). We evaluate the model prediction performance through cross-validation comparison. For dynamic gesture recognition, we investigate the recognition of 10 dynamic gestures using Hidden Markov Models (HMM) and Attention-Biased Mechanisms for Bidirectional Long- and Short-Term Memory Neural Network Models (Attention-BiLSTM). We analyze the differences in accuracy for complex dynamic gesture recognition with different feature datasets and compare them with the prediction results of the traditional long- and short-term memory neural network model (LSTM). Experimental results demonstrate that the random forest algorithm achieves the highest recognition accuracy and shortest recognition time for static gestures. Moreover, the addition of the attention mechanism significantly improves the recognition accuracy of the LSTM model for dynamic gestures, with a prediction accuracy of 98.3%, based on the original six-axis dataset.

## 1. Introduction

Gesture recognition is a topic in computer science and language technology [1]. As an extremely efficient non-verbal interaction method, gesture interaction will provide strong technical support for excellence in emerging fields such as smart medical devices, assistive devices for the disabled, smart homes, and smart military operations [2,3]. Most of the current major research work on gesture recognition is focused on machine-vision-based recognition methods, which can pose many limitations in practical applications. Inertial sensor-based gesture recognition is mainly focused on the improvement of recognition algorithms. This limits the application of gesture recognition algorithms in practical products [4,5]. The inertial sensor-based recognition method has an important role in helping to improve the accuracy of gesture recognition. Qiu et al. [6] introduced the devices and key applications of common wearable sensors, and discussed further research directions. It can be seen that wearable device technology could be the main research direction in gesture recognition research.

**Figure 1:**
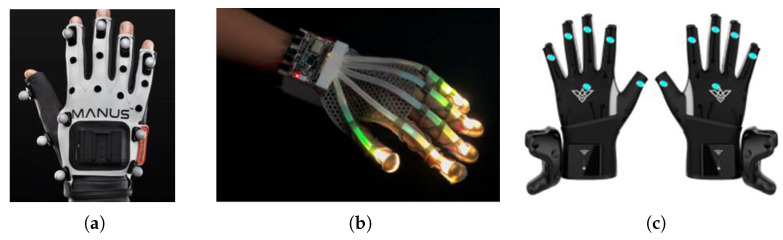
Mainstream data glove types. (**a**) Optical gloves (**b**) Fiber optic gloves (**c**) Inertia gloves.

Gesture recognition technology can be divided into static gesture recognition technology and dynamic gesture recognition technology according to whether it can recognize gestures based on time series [4]. At present, there are two main ways to collect gesture data, which are non-contact, based on machine vision sensors, and contact, based on data gloves [7]. The mainstream data-acquisition gloves are divided into three categories, as shown in Figure 1. Vision-based action recognition has been widely used and generally consists of four steps: gesture detection and segmentation, gesture tracking, feature extraction, and gesture classification [8]. Kinect [9] is a depth vision sensor released by Microsoft in 2010. Based on its built-in algorithm, it can automatically identify and track the dynamic skeletal structure of the human body and apply it to the hand to research human gestures. Researchers have two methods of gesture recognition using Kinect: (1) recognition based on the dynamic skeleton of the human body [10]; (2) recognition based on spatial depth sensing [5]. In the first approach, Ren et al. [11] obtained the skeleton data of 25 joint points of the human body by Kinect, obtained their coordinates in 3D space in real-time, and investigated the importance of each joint bone in dynamic gesture expression. However, they based this on the visual sensor to process the information of all parts of the whole body, and the gesture data were not expressed in enough detail. In the second approach, Wang et al. [12] obtained a higher accuracy rate by studying people’s gesture habits and the depth data from the Kinect depth sensor to control the 3D-printed robot. However, the results indicated that the recognition speed still needs to be improved. The leap motion sensor [13] is a somatosensory controller released by Leap in 2013. Unlike Kinect, it mainly performs skeletal motion tracking of the hand. Li et al. [14] generated finger-motion data with the help of Leap Motion Controller, which was used to calculate the angle of finger joints. This is a typical application of vision-based recognition; in addition to this, the research on inertial sensor-based recognition also has to be further improved.

The research on gesture recognition based on data gloves generally uses inertial sensors, myoelectric sensors, pressure sensors, and bending sensors on data gloves to obtain various gesture signals during hand movement. Alemayoh et al. [15] used inertial force sensors to capture motion data and then trained four neural networks using deep learning methods. The results showed that the vision transformer (ViT) network performed the best, with 99.05% recognition accuracy. Lin et al. [16] designed a data glove with multi-channel data transmission based on hand poses and emotion recognition to achieve simultaneous control of a robotic hand and a virtual hand. Zhao et al. [17] designed a motion-capture device based on a human sensor network with 15 sensor nodes and used the gradient descent method to fuse sensor data to improve the localization accuracy of the motion capture system. Liu et al. [18] proposed a novel gesture recognition device, which consists of a data glove with bending sensors and inertial sensors and a data arm ring with myoelectric sensors, to build a gesture recognition device. However, the sensors need to be used with electrode patches, which are very inconvenient to wear and replace; this is an area in urgent need of improvement. Fu et al. [19] proposed a gesture-recognition method based on a data glove and back propagation neural network. Only the gesture data of numbers 0–10 were used in the experiments, which lacked the recognition of dynamic gestures. Gałka J. et al. [20] introduced a construction of an accelerometer glove and its application in sign language gesture recognition. The basic data of inertial motion sensors and the design of gesture acquisition systems are also introduced. The solution presents the results of gesture recognition, selects a specific set of sign language gestures, and uses a description method based on the hidden Markov model (HMM) and parallel HMM methods. Using parallel hidden Markov models for sensor fusion modelling reduces the error rate by more than 60% while maintaining 99.75% recognition accuracy. Qiu et al. [21,22] used inertial sensors and data fusion algorithms to calculate the joint angles of kayakers, four machine learning algorithms were used to investigate the effect of different data combinations on phase classification, and extended Kalman filtering methods were used to fuse the sensor information all of which show good classification accuracy. Tai et al. [23] studied the continuous recognition of six types of gestures using smartphones combined with long- and short-term memory neural networks (LSTM), but the gesture actions used were too simple. The LSTM algorithm in [24] can be combined with convolutional neural networks for VGR-based gesture recognition; although these algorithms have been found to be effective, they still recognize only a single continuous gesture, and the problem of multi-class dynamic gesture recognition remains to be solved. Yuan et al. [25] design a wearable device with two arm loops and a data glove with integrated flexible sensors to capture fine arm and joint movements, and introduced an LSTM model with fused feature vectors as input to verify that the contextual information of gestures can be integrated in the gesture-recognition task and achieve excellent recognition results. Fan et al. [26] proposed a two-stage multi-headed attention human interaction action recognition model based on inertial measurement unit, which can accurately recognize seven interaction actions with an average recognition accuracy of 98.73%.

In order to recognize two types of gestures based on inertial sensors in indoor scenes, we propose recognition and analysis algorithms based on machine learning and deep learning. Applying traditional machine learning algorithms to static gesture recognition, we propose a bidirectional long- and short-term memory neural network model (attention-BiLSTM) based on the attention mechanism for the recognition study of 10 dynamic sign languages. The raw data are collected through a homemade data-collection glove and the gesture information is predicted using different machine learning algorithms. The aim was to improve the accuracy of gesture recognition and reduce the time of gesture recognition by building a gesture model, thus expanding the application scenarios of gesture recognition. The important contributions of this paper are as follows.

- The raw data were filtered using a Butterworth low-pass filter, the magnetometer data were corrected using an ellipsoidal fitting method, and the dataset was constructed using a gesture-assisted segmentation algorithm.
- We used four machine learning algorithms to identify static gesture data and evaluate the prediction effect by cross-validation.
- We constructed a hidden Markov model and an attention-based mechanism neural network model to design recognition methods for dynamic gestures.

This paper is structured as follows. Section 2 describes the hardware and data-acquisition information of the system. The research methodology is described in Section 3. The Section 4 shows the algorithm design of gesture recognition. Section 5 explains the results of this study. Finally, Section 6 shows the discussion and conclusion.

## 2. Systematic Data Collection and Participants

### 2.1. System Setup

**Figure 2:**
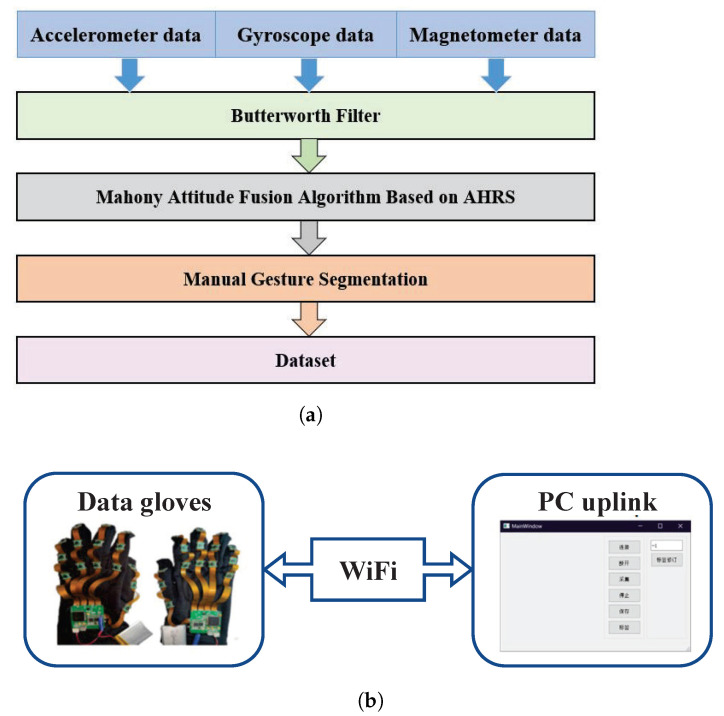
System configuration (**a**) gesture data collection and processing flow. (**b**) Gesture data acquisition system based on inertial sensor.

The gesture data acquired by the inertial sensors were processed by gesture segmentation, filtering and gesture fusion algorithms. The flowchart is shown in Figure 2a. A home-made data acquisition system was the main source of gesture data. The composition of the data acquisition system is shown in Figure 2. This is mainly composed of a pair of data gloves, a WiFi transceiver node and a personal computer (PC) host computer, which can complete the collection and storage of gesture data.

**Figure 3:**
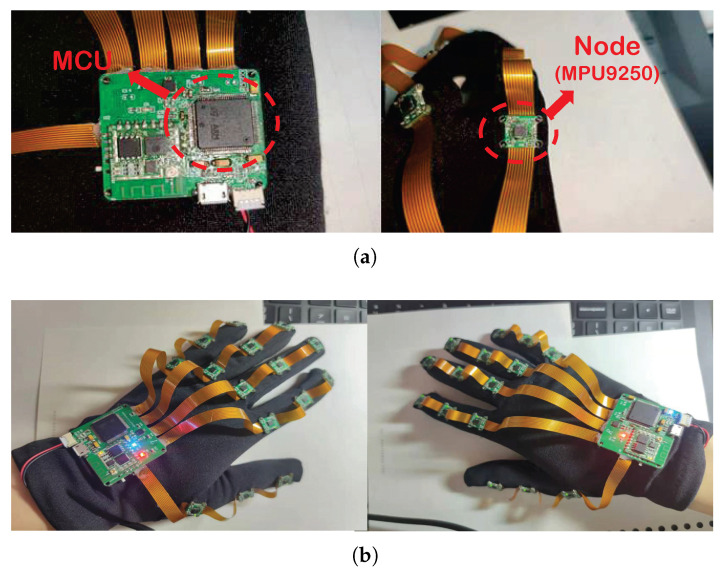
Physical hardware (**a**) sink and inertial nodes (**b**) wear display effect.

Each glove contains 16 inertial nodes, and the data collected by the inertial nodes can be sent to the PC host computer through the wireless module in real-time. The hardware part of the glove includes 15 inertial nodes and 1 sink node, as shown in Figure 3a. The aggregation node adopts STM32F407VGT6 Microcontroller Unit(MCU) as the main controller and is equipped with an ESP8266 serial peripheral interface bus (SPI) interface WiFi module produced by Espressif. The sampling frequency of the node was set to 100 Hz and the collected data can meet the needs of the gesture recognition algorithm. Because it uses wireless transmission, too high a sampling rate may increase the packet loss rate during transmission. The data of each sensor node are collected and processed by the sink node, and the PC receives them synchronously through WiFi. The positions of 15 inertial nodes correspond to 15 finger bones, which can detect the data of each finger. The convergence node is located on the back of the palm, which can detect the data of the palm. The glove material currently used has a certain degree of elasticity, and can increase the size of the internal volume within a certain range after the installation of sensors. Common hand sizes can basically meet the wearing requirements. The wearing effect is shown in Figure 3b.

**Figure 4:**
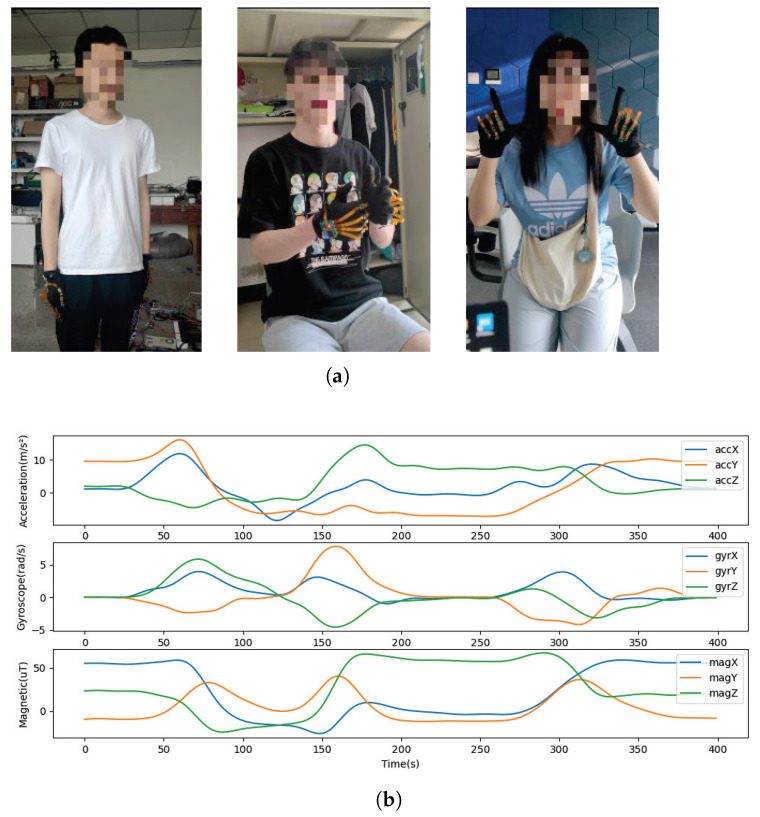
Raw data collection. (**a**) subjects wear data gloves for gesture data-collection experiments; (**b**) raw data collected by inertial nodes.

The inertial node consists of MPU9250 9-axis sensors: a 3-axis accelerometer, a 3-axis gyroscope, and a 3-axis magnetometer. Taking node 1 of the dynamic one-hand gesture “Sorry” as an example, the obtained nine-axis raw data are shown in Figure 4b. All gesture data were obtained from the author and classmates wearing data gloves and completing the specified gestures. Prior to data acquisition, we had to perform initial calibration, i.e., calibration of the subject’s hand position by specific movements, due to the variation in glove-wearing position between participants, as well as the effects of the system’s duty cycle and the external environment. When collecting gestures, the person being collected faces due north, and the initial gesture posture is naturally drooping. When the system starts acquisition, the upper-computer data information can be observed, and after 3 s the acquisition system completes the coordinate system calibration to facilitate the conversion of gesture data. During the subsequent acquisition actions, the collector can face in any direction. The gesture collection is shown in Figure 4a.

In this paper, we used different recognition classification algorithms for static gesture and dynamic gesture recognition. Static gestures are hand patterns that are fixed at a certain moment in time, and each frame captured by the data glove can be used as a set of gesture samples. A large amount of information about dynamic features can be removed when performing feature extraction, and then traditional machine learning algorithms are used for gesture recognition. Specifically, these include: support vector machines, back-propagation neural networks, decision trees and random forest model algorithms. The dynamic gesture features are obviously more complex than static gestures, with the sign language actions changing all the time and the execution time varying between sign languages. Therefore, we used HMM- and attention-BiLSTM-based bi-directional long- and short-term memory neural network models for the recognition study of 10 dynamic sign languages, and the latter served to validate dynamic sign language recognition on deep learning methods.

### 2.2. Participant and Gesture Acquisition Actions

Four students recruited from the school participated in the preliminary study. Their average weight was 70.5 ± 2.3 kg and their average height was 1.74 ± 0.58 m. The participants consisted of three male students and one female student, and each student sampled 50 sets of gesture data, for a total of 200 samples per gesture. All participants recorded their height and weight, and received adequate information, and particpants’ consent was obtained.

A total of 20 static-letter gestures and 10 dynamic one-handed gestures were selected for this study. Twenty static-letter gestures were as follows: “A”, “C”, “D”, “E”, “F”, “G”, “H”, “K”, “L”, “M”, “N”, “O”, “Q”, “R”, “S”, “U”, “V”, “W ”, “X”, “Y”. Ten dynamic one-handed gestures were as follows: “Sorry”, “Angry”, “Sad”, “You”, “Hello”, “Effort”, “They”, “Me”, “Thanks”, “Goodbye”. All sign standards are referenced in the “Sign Language Dictionary | SpreadTheSign” page. Chinese sign language standards are used.

## 3. Methods

### 3.1. Definition of Coordinate System and Conversion Relationship

The gesture recognition system will involve conversion between common coordinate systems and multiple coordinate systems. The measurement values of the sensor nodes can be converted to the geographical coordinate system by means of the conversion relationships between different coordinate systems. In this system, three coordinate systems are included and each coordinate system is based on the standard right-handed 3D Cartesian coordinate system. Detailed information is as follows.

- Sensor coordinate system (SCS): typically, the origin of the sensor coordinate system is the sensor center, and the pointing of the three axes is based on the three-axis gyroscope in the sensor.
- Navigation coordinate system (NCS): the origin is the center of gravity of the hand when standing, and the three axes point to the northeast and the ground direction. It should be noted that north here refers to the north in the geomagnetic sense.
- Body coordinate system(BCS): according to the spatial posture of the palm and each finger segment, the center of mass of the hand is used as the origin of coordinates.

In the initial state, the data are collected facing north so that the sensor coordinate system and the body coordinate system coincide, which facilitates gesture posture calculation and spatial coordinate conversion [17].

At present, the automatic heading reference system (AHRS) attitude calculation algorithm is the main means of aircraft attitude calculation [27]. Since the spatial description of the gesture node has similar characteristics to the spatial description of the aircraft, Euler angles and quaternions are also used to describe the gesture attitude. Quaternions are defined as follows, where *i*, *j*, *k* are the standard orthonormal basis for unit vector representation in 3D space.

(1) $$q=q_{0}+q_{1}\vec{i}+q_{2}\vec{j}+q_{3}\vec{k}$$

In the actual gesture data acquisition process, the motion data output from inertial nodes, including acceleration, angular velocity, and magnetic field strength, correspond to SCS. We need to convert the sensor output to gesture pose under NCS. This can be achieved by matrix changes between coordinate systems. Let $C_{B}^{n}$ serve as the pose transformation matrix from the sensor coordinate system to the navigation coordinate system, and $C_{B}^{n}$ can be expressed as a quaternion.

(2) $$C_{B}^{n}=\left[\begin{array}{ccc} 1-2\left(q_{2}^{2}+q_{3}^{2}\right) & 2(q_{1}q_{2}+q_{0}q_{3}) & 2(q_{1}q_{3}-q_{0}q_{2})\\ 2(q_{1}q_{2}-q_{0}q_{3}) & 1-2\left(q_{1}^{2}+q_{3}^{2}\right) & 2(q_{2}q_{3}+q_{0}q_{1})\\ 2(q_{1}q_{3}+q_{0}q_{2}) & 2(q_{2}q_{3}-q_{0}q_{1}) & 1-2\left(q_{1}^{2}+q_{2}^{2}\right) \end{array}\right]$$

The relationship between the attitude angle and the quaternion can be expressed as follows:

(3) $$\left\{\begin{array}{c} \theta =\arcsin\left[2\left(q_{0}q_{2}-q_{1}q_{3}\right)\right]\\ \gamma =\arctan\left(\frac{q_{0}q_{3}+q_{1}q_{2}}{1-2\left(q_{2}^{2}+q_{3}^{2}\right)}\right)\\ \psi =\arctan\left(\frac{q_{0}q_{1}+q_{2}q_{3}}{1-2\left(q_{1}^{2}+q_{2}^{2}\right)}\right) \end{array}\right.$$

where γ, θ, ψ represent the cross roll angle, pitch angle and heading angle, respectively.

**Figure 5:**
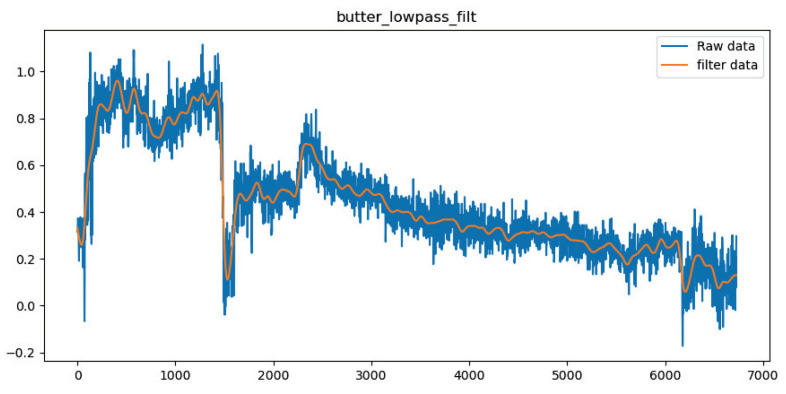
Raw data filtering effect.

The inertial measurement unit (IMU) sensor needs to be filtered due to the high level of noise. The that remain data after removing the noise are used for attitude-solving. In this paper the raw data are processed using Butterworth filter, which is a signal processing filter also known as the maximum flat filter. Its filtering effect is shown in Figure 5.

### 3.2. Calibration of Magnetometer Based on Ellipsoidal Fitting Method

**Figure 6:**
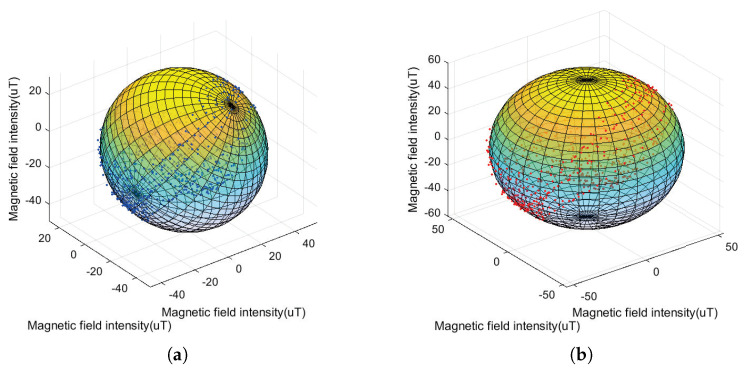
The calibration outcome of magnetometer. (**a**) before fitting. (**b**) after fitting.

At the beginning of the gesture-recognition process, the magnetometer needs to be calibrated due to the presence of hard iron distortion and soft iron distortion in the surrounding environment. The main sources of hard iron distortion are permanent magnets and magnetized metals. To eliminate the ferromagnetic interference, the ellipsoidal fitting method is used to eliminate the ferromagnetic interference. Soft iron is relatively small and negligible [28]. We used the eight-character calibration method and the single-axis rotation method for magnetometer calibration. That is, after the gesture data acquisition system works, it is rotated around the figure of eight three times and then rotated along each single axis three times. The ellipsoidal fitting results are shown in Figure 6. As can be seen from the figure, before the magnetometer calibration, the measured values are disturbed by the surrounding environment, the fitted ellipsoidal spherical center is not located at the coordinate origin and the sphere is tilted. After the calibration, the center of the fitted sphere is located at the origin and the position is accurate.

### 3.3. Gesture Dataset Segmentation

**Figure 7:**
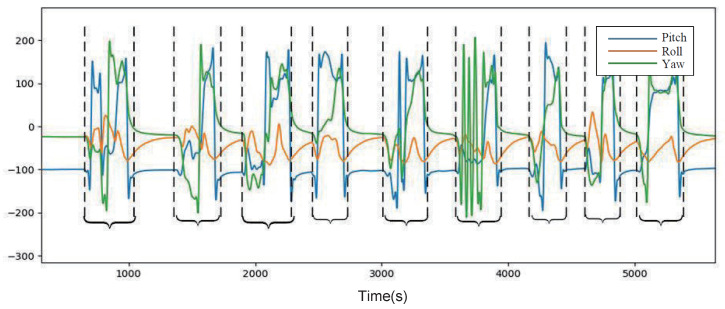
Dynamic gesture data segmentation.

The segmentation process of the gesture dataset is as follows. The average of every 50 sets of static gesture data is used as one sample. The sampling frequency is set to 100 Hz, i.e., the average of every 0.5 s of data is used as one gesture sample. Since the task is dealing with time-series-based gesture classification, the sliding window method is used for segmentation, and a total of 200 sets of samples are obtained for each gesture. The length of the frontal sequence of the sliding window is defined, and the valid gesture data are intercepted sequentially starting from the starting point. The detailed data segmentation algorithm is shown in the Algorithm 1. The dynamic gesture raw data consist of two parts, transition gestures and valid gestures, as shown in Figure 7.
**Algorithm 1:** Assisted Segmentation Algorithm**Input**: Continuous gesture data: $D=\left\{d_{1},d_{2},d_{3},...,d_{n}\right\}$;**Output**: Split start point list *S*, split end point list *O*.1Transition state mean: $m=\frac{\sum_{i=1}^{100}d_{i}}{100}$;2Define the length l of the sliding window sequence, the current window value $A_{0}$, the window value $A_{1}$ after sliding 5 times, the window value $A_{2}$ after sliding 10 times, $E_{1}=\left|A_{0}-A_{1}\right|$,$E_{2}=\left|A_{1}-A_{2}\right|$;3Traverse continuous gesture data D;4**if** $\left(E_{1}>10\right)\&\&\left(\left|A_{0}-m\right|<12\right)\&\&\left(\left|A_{2}-m\right|<12\right)$ **then** Read the current position and add it to the starting point list S; (5**if** $\left(E_{2}>10\right)\&\&\left(\left|A_{0}-m\right|<12\right)\&\&\left(\left|A_{2}-m\right|<12\right)$ **then** Read the current position and add it to the end point list O; (6End;

## 4. Design of Gesture Recognition Algorithm

### 4.1. Static Gesture Recognition Method Based on Machine Learning Algorithm

**Figure 8:**
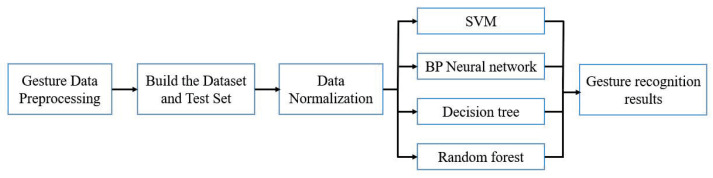
Static gesture recognition flowchart.

This section introduces the implementation of the four machine learning algorithm models for gesture recognition. The specific flowchart is shown in Figure 8.

Static gesture refers to the static spatial pose of the hand, and most sign languages convey meaning using the static pose of the hand in the air. For example, thumbs up means “good”. In this process, it is not necessary to reflect the change in the hand posture with time and space; only the static posture of the hand is discussed. The original data collected by the data glove are the data of the 16-node three-axis accelerometer, three-axis gyroscope and three-axis magnetometer. The original data structure is as follows:

(4) $$RawData=\left[ax_{i},ay_{i},az_{i},gx_{i},gy_{i},gz_{i},mx_{i},my_{i},mz_{i}\right],i\in \left[1,16\right]$$

After filtering and attitude fusion algorithm processing, each group of gesture data are composed of Euler angle data of 16 nodes. The data structure is as follows:

(5) $$StaticGesture=\left[yaw_{i},roll_{i},pitch_{i}\right],i\in \left[1,16\right]$$

1. Support Vector Machines
  SVM [29] is a binary classifier based on supervised learning first proposed by Corinna Cortes and Vapnik et al. in 1995, whose decision boundary is the maximum margin hyperplane solved for the learned samples.
  The grid search method is an optimization method of parameter selection and cross-validation by specifying a selection list of parameters to be optimized, evaluating the model for all parameter combinations, and finally obtaining the optimal parameters in the list. This method is used in the experimental parameter optimization. The structure of the dataset used is as follows: Training set: $TrainDataSet=\left\{D{\left(People_{1}\right)}_{i},D{\left(People_{2}\right)}_{i}\right\},i\in \left[1,70\right]$. Test set: $TestDataSet=\left\{D{\left(People_{1}\right)}_{j},D{\left(People_{2}\right)}_{j}\right\},j\in \left[71,100\right]$. The training set part is used for algorithm model training, and the test set data are used to test the algorithm recognition accuracy of the generated model.
2. Back-Propagation Neural Network
  Neural network (NN) [30] is a mathematical model or computational model that simulates the structure of biological nerve cells to receive stimulation and generate output signals, and simulates the excitation function, as shown by Michael Houston et al.
3. Decision Tree algorithm
  Decision tree (DT) is a common classification algorithm based on supervised learning in machine learning. Its advantages are its simple structure, logic in line with human intuition, and fast processing speed for large amounts of data. In this paper, we use “information entropy” and “Gini index” to classify the attributes for model training. The CART algorithm and ID3 algorithm have overfitting problems, and the generalization ability of the model can be improved by discarding the over-divided attributes.
4. Random forest algorithm
  Random forest (RF) is an ensemble algorithm consisting of multiple decision trees. The random forest is composed of several decision trees, and the Gini index is better than the information entropy in dividing conditions with judgmental attributes, so the Gini index is also used to construct the random forest to prevent model overfitting. The maximum depth of the tree is defined as 6 and the number is 20.

### 4.2. Dynamic Gesture Recognition Based on Hidden Markov Model

**Figure 9:**
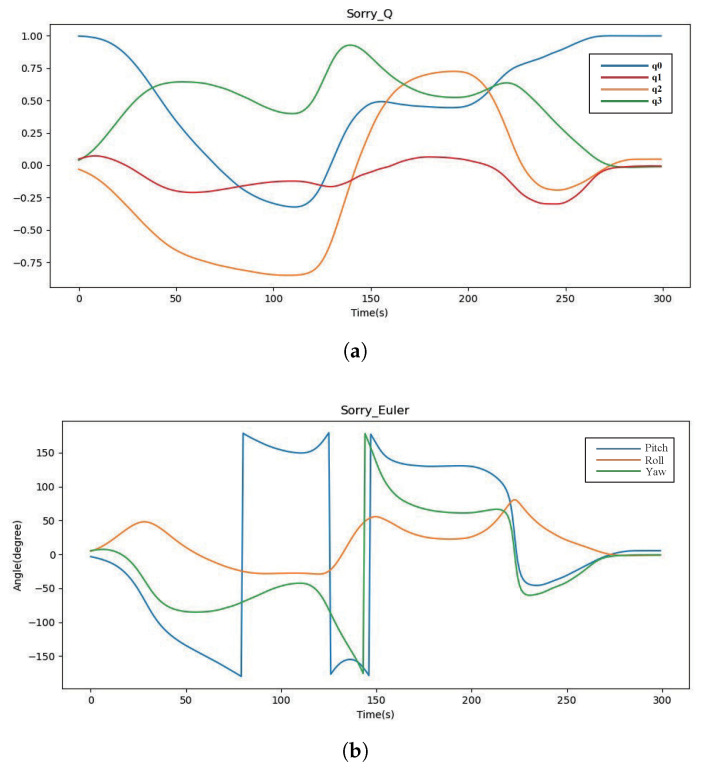
Dynamic gesture “Sorry” data graph. (**a**) Quaternion data; (**b**) Euler angle data.

Dynamic gestures are action sequences composed of gesture transformations in space based on time sequences. Richer and more complex gesture information can be expressed. Different datasets express the gesture features in different ways, and the raw data contain all the gesture information, in addition to valid data, as well as noise and interference. The Eulerian angle dataset better describes the gesture changes in gesture motion. Quaternions can solve the shortcomings of Euler angles in gesture description. Each of the three data structures is shown below:

(6) $$R\mathrm{aw}Data_{t}=\left[ax_{i_{t}},ay_{i_{t}},az_{i_{t}},gx_{i_{t}},gy_{i_{t}},gz_{i_{t}},mx_{i_{t}},my_{i_{t}},mz_{i_{t}}\right],i\in \left[1,16\right]$$

(7) $$\left\{\begin{array}{c} DynamicGestureE_{t}=\left[yaw_{i_{t}},roll_{i_{t}},pitch_{i_{t}}\right],i\in \left[1,16\right]\\ DynamicGestureQ_{t}=\left[q1_{i_{t}},q2_{i_{t}},q3_{i_{t}},q4_{i_{t}}\right],i\in \left[1,16\right] \end{array}\right.$$

where *DynamicGestureE* represents Euler angular data and *DynamicGestureQ* represents quaternion data. Taking the one-handed dynamic gesture “Sorry” as an example, the preprocessed quaternion and Euler angle convergence node (node1) data are shown in Figure 9a,b, respectively.

Hidden Markov model (HMM) [31] is a statistical model created in the 1970s. Hidden Markov model is a kind of Markov chain (MC) [32], which is the simplest dynamic Bayesian network. HMM is effective in solving time-series-based data-recognition problems, and the dynamic-gesture recognition problem is a strong time-series-related recognition problem. In the gesture-recognition task, the feature data of each node are a visible observation sequence, and the meaning of the gesture transition is an unknown state sequence. HMM can generate gesture transition-state sequences based on the observed sequence of gestures and generate recognition models.

Hidden Markov models can be used in prediction problems: from a determined sequence of observations, a sequence of states is obtained computationally. For example, in the gesture recognition problem, the state is the meaning of the gesture and the observation is the sequence of gesture actions. The algorithm is generated as follows:

- We generate the first gesture meaning given the initial state probability vector π;
- According to the previous gesture meaning, the next gesture meaning is randomly generated using the state transfer probability matrix A;
- After generating the sequence of gesture meanings, the observation sequence of the corresponding position is generated using the observation probability matrix B according to each gesture meaning.

**Figure 10:**
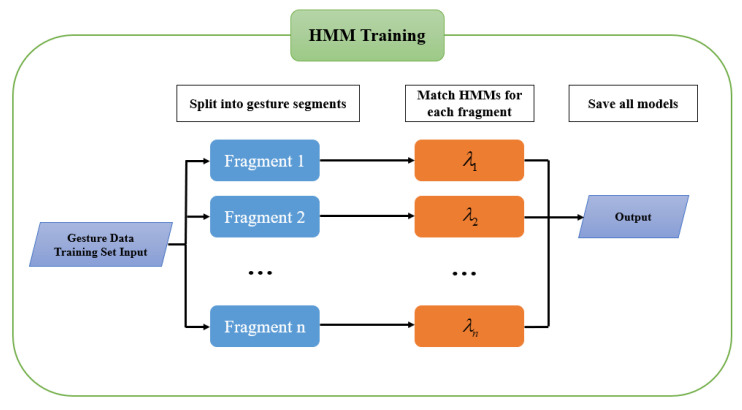
HMMs training flow chart.

In the gesture-recognition problem, we can train the model by studying the recognition problem. The HMM training process is shown in Figure 10.

First, the training set of gesture data is used as input, and divided into gesture segments that need to be recognized. Each gesture segment corresponds to a gesture semantics, and the gesture semantics are marked and trained. Each individual semantic needs to train a corresponding hidden Markov model, train the model parameters with the highest matching degree through the specified hidden Markov model structure, and finally save all the models. This process can be solved using the Baum–Welch algorithm.

**Figure 11:**
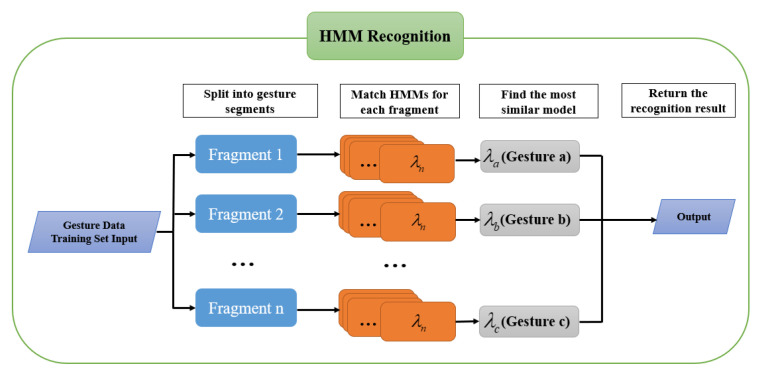
HMMs identification flow chart.

The best hidden Markov model corresponding to each gesture category is obtained by solving the training problem, and the classification problem of gesture recognition can be solved by solving the evaluation problem. The evaluation problem uses a new observation sequence $O^{\prime }=\left({o^{\prime }}_{1},{o^{\prime }}_{2},\ldots ,{o^{\prime }}_{n}\right)$. The probability $P\left(O^{\prime };\lambda \right)$ of producing this observation sequence $O^{\prime }$ must be found. The process of dynamic gesture recognition is shown in Figure 11.

Then, we use the forward–backward algorithm to solve the unknown gesture-classification problem. The gesture category corresponding to the model with the highest matching degree is selected as the prediction result.

### 4.3. Design of Gesture-Recognition Algorithm Based on Deep Learning

**Figure 12:**
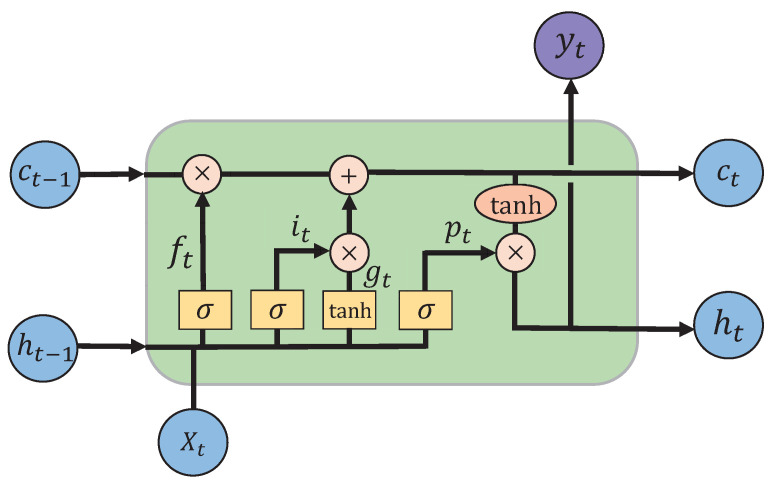
LSTM structure diagram.

Long short term memory neural network (LSTM) is a recurrent neural network model proposed by Hochreiter and Schmidhuber in 1997 [33], whose single-neuron structure is shown in Figure 12.

**Figure 13:**
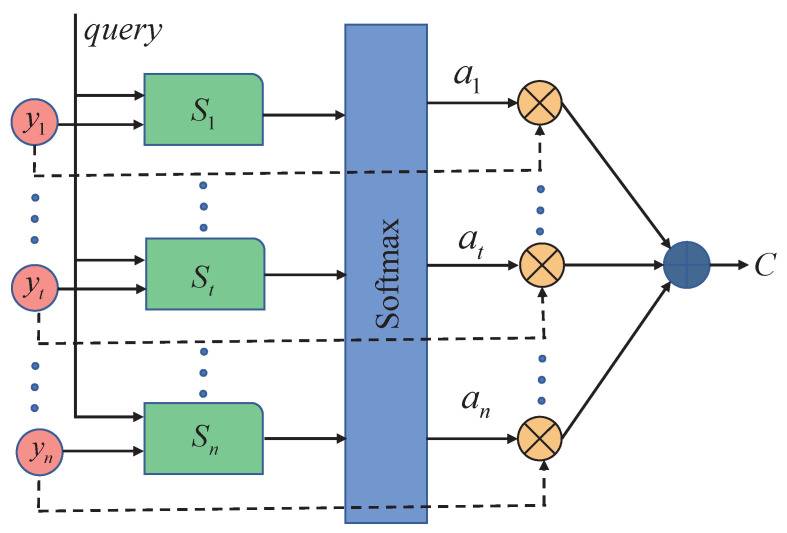
Structure of attentional mechanisms.

However, such recurrent neural network models can only perform derivation and learn information before that moment in a unidirectional way, and are unsuitable for more refined recognition, such as textual information and time sequences. We introduce a bi-directional long short term memory neural network (Bi-LSTM), which can learn the bi-directional information of sequences. The Bi-LSTM model adopts the traditional encoding–decoding method, and sequence samples are input to Bi-LSTM and edited into a fixed-length vector representation regardless of their length. Although Bi-LSTM can memorize the forward and backward data information, in practical applications, due to the differences in sequence lengths of different samples, it may appear that certain key factors are overlooked when training the model, resulting in model-recognition performance degradation. In response to its characteristics, an attention mechanism is introduced, which essentially imitates a person observing something important in a scene and focusing his or her attention on that part [34]. Introducing the attention mechanism can break the problem of fixed vector length in the encoding process of Bi-LSTM, and provide the corresponding weights according to the characteristics of the sequence to show the key information more clearly, which can improve the model training efficiency and help the model in accurate recognition. The structure diagram is shown in Figure 13, where $y_{i}$ is the hidden layer vector output by Bi-LSTM at each moment as key, and $y_{n}$ output at the last moment as query, which is calculated as follows:

(8) $$S_{t}=\alpha \left(y_{t},y_{n}\right)$$

(9) $$a_{t}=\frac{\exp(S_{t})}{\sum_{t=1}^{n}\exp\left(S_{t}\right)}$$

(10) $$c=\sum_{t=1}^{n}a_{t}y_{y}$$

where $S_{t}$ is the similarity score between $y_{t}$ and $y_{n}$ at each moment calculated by the learning function α; then, it is normalized by the softmax function to obtain the weight $a_{t}$ of $y_{t}$ at each moment, and finally the vector c is calculated by Equation (10).

**Figure 14:**
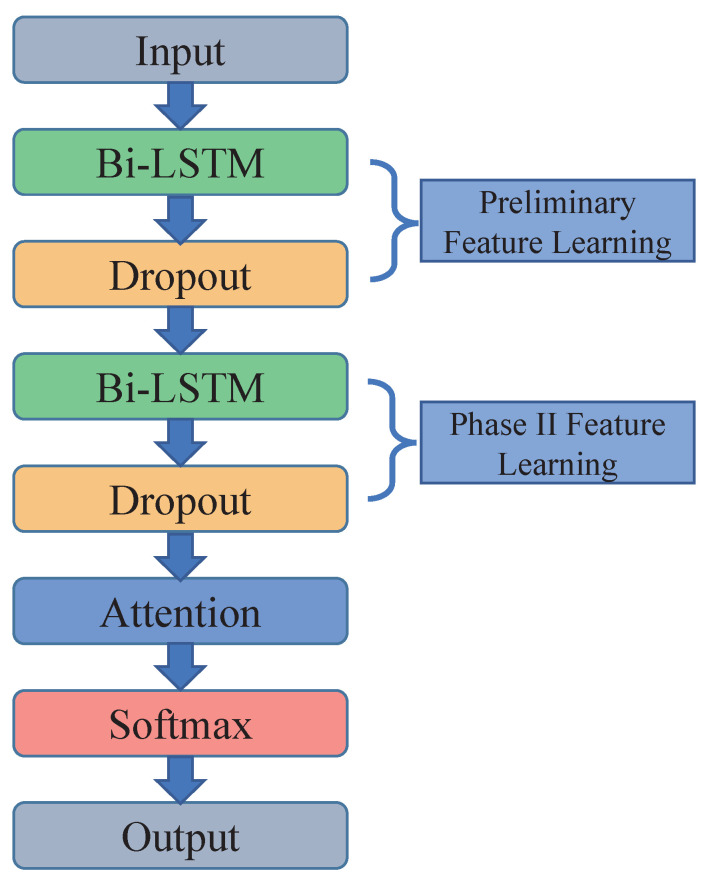
Attention-BiLSTM model structure.

Considering that the sign language data consist of time series of acceleration, angular velocity, and pose quaternions, and the dataset is small, Bi-LSTM can better learn features from the contextual information of sign language time series. Finally, we designed the Bi-LSTM, which introduces the attention mechanism (attention-BiLSTM), and its overall structure is shown in Figure 14. Firstly, the collected sign language acceleration, gyroscope and pose quaternion data are passed into the input layer, and initial feature learning is performed by the Bi-LSTM layer for sign language information; then, the data are input to the lower Bi-LSTM layer through the Dropout layer for the second stage of feature learning, after which the similarity is calculated by passing the dropout layer into the attention layer. Finally, the softmax layer normalizes these, further calculates the weight information of each moment, and sends the result to the fully connected layer. The output layer gives the sign language recognition result.

## 5. Results

We performed static and dynamic gesture-recognition tasks to verify the recognition performance of the machine learning and deep learning algorithms used, and provide detailed recognition results for the two types of gestures under different algorithms.

### 5.1. Static Gesture Recognition Using Machine Learning Algorithms

1. Support Vector Machines

When building a support vector machine model, the kernel functions are first selected and the accuracy of all kernel functions is tested. The multiclassification SVM model built using the one vs. rest (OvR) strategy. The kernel function test results are shown in Table 1.

2. BP Neural Network

Due to the low complexity of static gesture data and to prevent overfitting, a neural network structure with 2 hidden layers and 20 neurons per layer was built. The prediction accuracy of different activation functions was tested by the grid optimization algorithm, as shown in Table 2.

3. Decision Tree algorithm

Generally, the most important part in deciding the superiority of decision tree classification is the judgment algorithm of attribute division. Information entropy and the Gini index are used for model training, and the accuracy is shown in Table 3.

4. Random Forest algorithm

**Figure 15:**
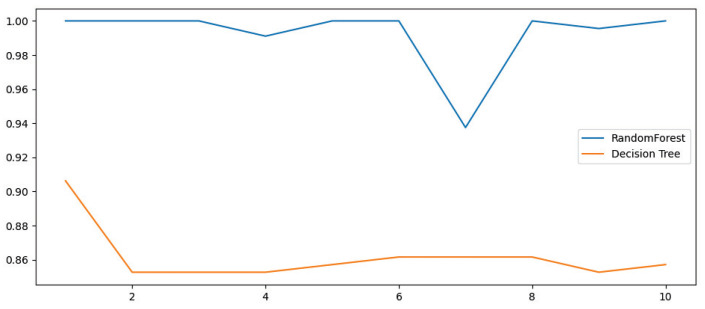
Random Forest vs. Decision Tree Cross-Validation.

We define the maximum depth of the tree as 6 and the number as 20. The random forest and decision tree are cross-validated 10 times, and the validation effect is shown in Figure 15.

**Figure 16:**
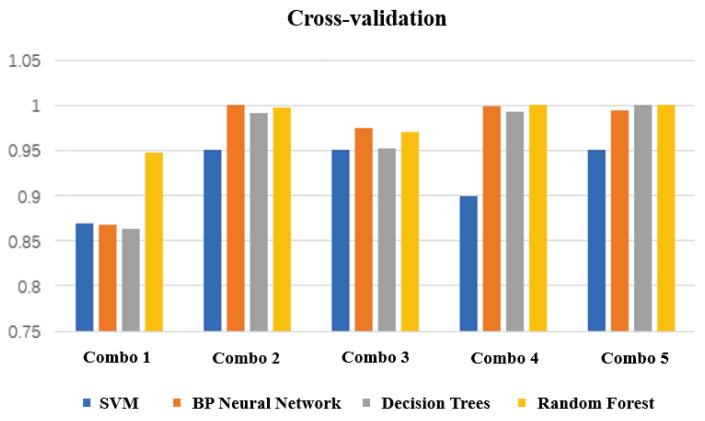
Cross-validation results.

After the model is built and trained, to evaluate the fit of the model, data outside the training set needs to be put into the model for evaluation, i.e., the test set. In order to better test the model, the idea of cross-validation is used to evaluate the model. We use 5-fold cross-validation, that is, the training set is divided into 5 equal parts, 5 unrelated subsets are obtained, and each subset is taken out in turn as the test set. The remaining 4 subsets are used as the training set, and cross-validation is repeated 5 times to obtain 5 test results. The final prediction results can be obtained by averaging the results, and the cross-validation results of each combination are shown in Figure 16. The final results of each model prediction are shown in Table 4.

From the table, it can be seen that the Random Forest algorithm has the highest accuracy rate and the shortest training time for static gestures, while the SVM algorithm has the lowest accuracy rate and the longest training time. This finding is also consistent with the cross-validation results.

### 5.2. Dynamic Gesture Recognition Based on Hidden Malcove Model

**Figure 17:**
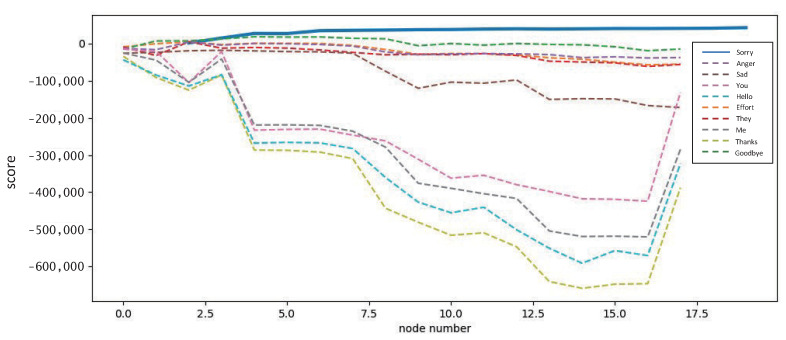
The number of nodes and the degree of model fit.

Although the hidden Markov model can be parameterized by the Baum–Welch algorithm, the priority needs to be determining the number of states for each hidden Markov model. Different dynamic gestures have different complexities, and choosing a suitable number of states according to a specific complexity can not only minimize the training time, but also improve the degree of model fitting and prevent the overfitting phenomenon that occurs with too many nodes. We build the model with different number of nodes, train it with the training set, and then test the model output with random samples from the test set. The results are shown in Figure 17, and the model fits and differentiates well when the number of nodes is 13.

**Figure 18:**
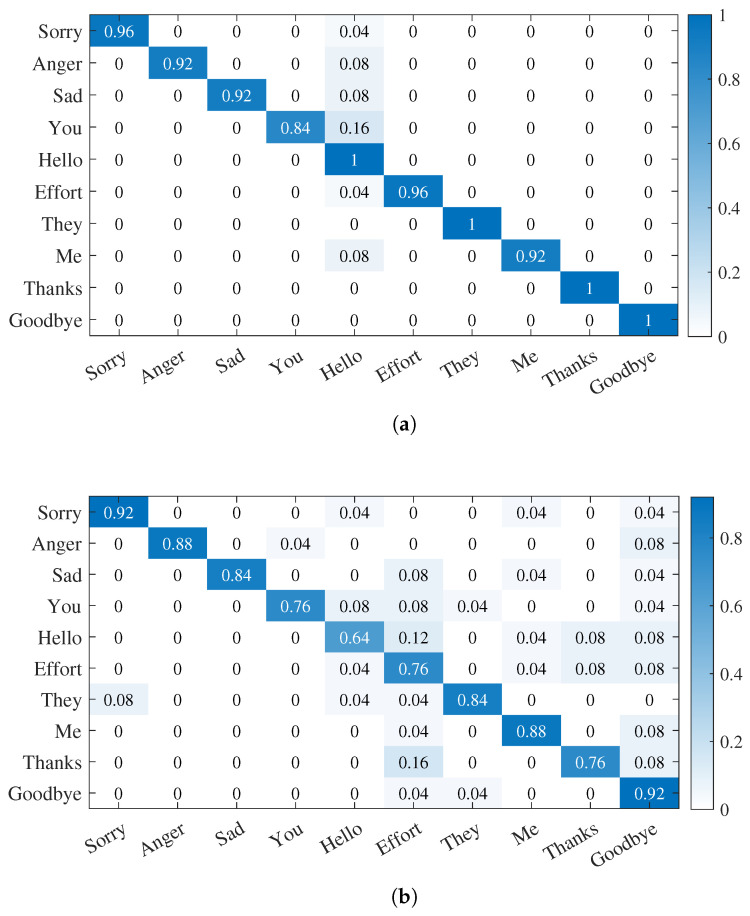
Confusion matrix results based on HMM. (**a**) Confusion matrix for gesture recognition based on six-axis data. (**b**) Effect of gesture recognition based on quaternions.

We trained and evaluated the models on the original dataset and quaternion dataset, respectively. The experimental results are shown in Table 5. It can be observed that the prediction model based on the original six-axis data achieves the highest recognition accuracy. However, as the raw data contain all features of dynamic gesture data, the training and testing times of the model are long. Therefore, it is necessary to extract features from the original data, remove invalid features, or reduce the dimensionality of the data. The confusion matrix obtained for different datasets is shown in Figure 18.

### 5.3. Dynamic Gesture Recognition Based on Deep Learning Methods

**Figure 19:**
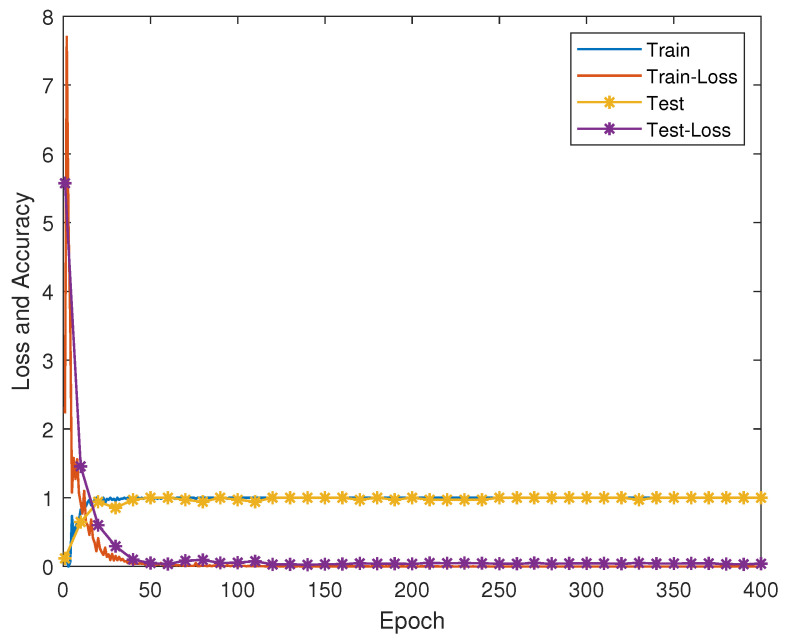
Model training process.

**Figure 20:**
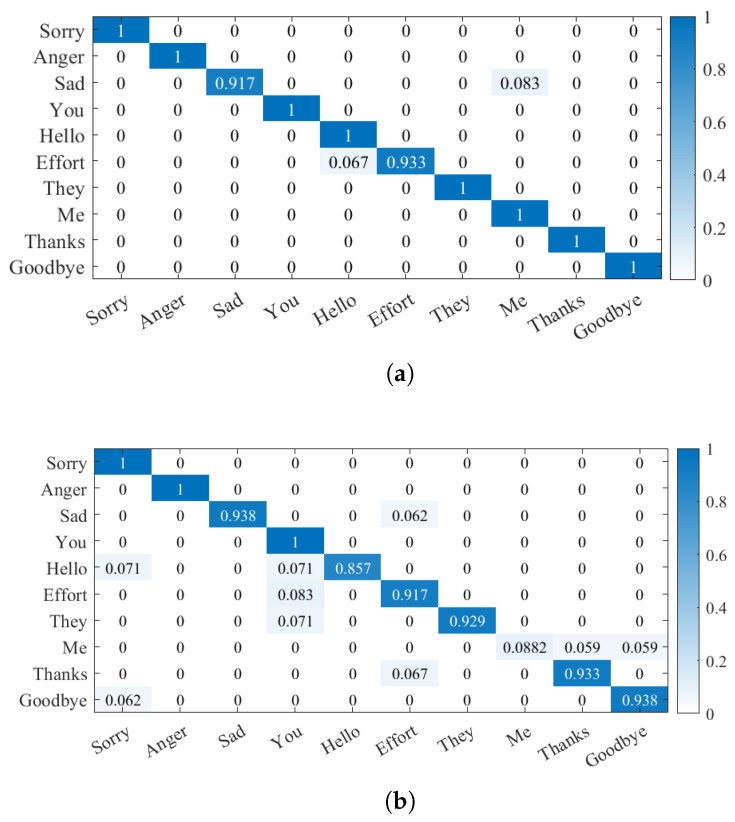
Confusion matrix results based on Attention-BiLSTM. (**a**) 6-axis data. (**b**) Quaternion data.

As the deep learning model may cause underfitting or overfitting problems due to the small training dataset or the existence of sample imbalances, we can configure the following parameters for this experiment. In the attention-BiLSTM model, we set the learning rate to 0.001, the training sample batch size to 256, and the number of iterations to 50. The training process, testing process, and loss function of the final model are shown in Figure 19. The training process of the model and the testing process of the loss function finally converge. We used 6-axis data and quaternion data for the recognition of 10 dynamic gestures, respectively, with 98.3% and 94.6% recognition accuracy when fed into a pre-prepared dataset that was not involved in training and validation. The confusion matrix of the model is shown in Figure 20. In comparison with the hidden Markov model, the bidirectional long- and short-term memory neural-network model based on the attention mechanism achieves better recognition results. It is worth noting that we also conducted recognition tasks for 10 dynamic gestures using the LSTM model, and the results are presented in Table 6. The addition of attention led to a significant improvement in gesture-recognition accuracy, which can be attributed to our effective utilization of both pre- and post-moment information in the gesture sequence.

## 6. Discussion and Conclusions

Gesture-recognition technology can be applied to many scenarios, such as virtual reality, robot control, and remote operation. The main sensors currently used regarding gesture recognition are IMU, video-based optical capture, and surface electromyography sensors. The main problems are the inconvenience of wearing and the vulnerability to environmental interference. Some studies focus on the structural design of the data glove and ignore the influence of the recognition algorithm on the recognition accuracy.

In this paper, an inertial sensor-based gesture data acquisition system is used, with the goal of constructing a gesture-recognition model based on the collected static and dynamic gesture datasets. Traditional machine learning algorithms can perform gesture recognition classificatio. We evaluated the prediction effectiveness of four algorithms. For static gestures, the model prediction performance was evaluated by cross-validation comparison, and we obtained the conclusion that the random forest algorithm has the highest recognition accuracy and the shortest recognition time. For dynamic gestures, we used HMM and a deep-learning-based attention-BiLSTM model and, according to the results, the latter achieved a higher recognition accuracy. The model can integrate the time series information of sign language acceleration, angular velocity, and hand posture to predict the sign language category, introduce a dropout layer to avoid model overfitting, and use the Adam optimization algorithm to accelerate the model convergence speed.

However, this does not indicate that deep learning methods are superior to traditional machine learning algorithms in the field of gesture recognition. This is because we can obtain a more comprehensive understanding of the data and the underlying algorithm of the model compared to the black-box structure of the deep model. Finally, in the field of practical engineering, traditional machine learning methods often require much less computational cost than deep learning methods. In the direction of gesture recognition based on wearable devices, it is often necessary to consider portability, power consumption, cost, comfort, etc. In the case of the compatible consideration of these factors, it is difficult to add the computational units required for deep learning, so it is difficult to determine the performance of deep learning models. In contrast, traditional machine learning models are fast to train, simple to deploy, and the required engineering costs are concentrated on data processing and feature optimization in the pre-model, thus allowing for faster update iterations in hardware products and the ability to try different model approaches in a short period of time. These aspects are not attainable by deep learning at this stage.

In addition, participants believed that prolonged wear would also cause hand discomfort. In this case, there is an urgent need for more comfortable gesture-recognition monitoring solutions or the use of fewer miniature inertial sensor nodes with guaranteed recognition performance. There were no strict criteria for the gesture data collected in this study, and the participants had no experience with gesture learning. These factors are worth considering in the future. In the future, we will consider designing more lightweight, miniature wearable device modules that can be integrated into existing electronics, such as watches and rings, to create a more comprehensive gesture-capture interaction system.

## Figures and Tables

**Table 1:** Kernel function accuracy.

Name	Experssion	Prediction Accuracy
Linear Kernel	$K\left(x,z\right)=x^{T}z$	98.12%
polynomial kernel	$K\left(x,z\right)={\left(x^{T}z\right)}^{d}$	96.96%
Gaussian kernel	$K\left(x,z\right)=\exp\left(-\frac{{∥x-z∥}^{2}}{2\sigma^{2}}\right)$	98.57%
Sigmoid Kernel	$K\left(x,z\right)=\tanh\left(\beta x^{T}z+\theta \right)$	28.93%

**Table 2:** Common activation functions and prediction accuracy.

Activation Function Name	Expression	Prediction Accuracy
Identity	$f\left(x\right)=x$	98.83%
Logistic	$f\left(x\right)=\frac{1}{1+e^{-x}}$	84.67%
Tanh	$f\left(x\right)=\tanh\left(x\right)$	92.58%
Relu	$f\left(x\right)=\max\left(0,x\right)$	95.92%

**Table 3:** Common discriminant algorithms and prediction results.

Discriminant Algorithm	Prediction Accuracy
Entropy	73.08%
Gini index	88.42%

**Table 4:** Model accuracy rate and training time.

Model Name	Accuracy Rate	Training Time (s)
SVM	92.4%	1.616
BP Neural Network	96.7%	0.249
Decision Trees	95.9%	0.011
Random Forest	98.3%	0.064

**Table 5:** Recognition accuracy and training time based on HMM.

Data Characteristics	Prediction Accuracy	Training Time(s)
Six-axis data	95.6%	518
Quaternions	82.1%	239

**Table 6:** Comparison of gesture recognition results based on attention-BiLSTM and LSTM models.

Data Characteristics	Attention-BiLSTM	LSTM
Six-axis data	98.3%	83.8%
Quaternions	94.6%	87.8%

## Data Availability

We have placed some of the gesture data used in the repository below. https://github.com/DUTRB/gestureData.

## Abbreviations

The following abbreviations are used in this manuscript:

AHRS	Attitude and heading reference system
BCS	Body coordinate system
BP	Back-propagation neural network
Bi-LSTM	Bidirectional long- and short-term memory neural network
CNN	Convolutional neural network
DT	Decision tree
HMM	Hidden Markov model
HOG	Hitogram of gradient orientation
HD-SEMG	High-density surface electromyography
IMU	Inertial measurement unit
LSTM	Long- and short-term memory neural network
MCU	Microcontroller unit
MEMS	Micro-electro-mechanical System
MC	Markov chain
NCS	Navigation coordinate system
NN	Neural networks
OvR	One vs. rest
PC	Personal computer
RF	Random forest
RNN	Recurrent neural networks
ROI	Region of interest
SPI	Serial peripheral interface bus
SCS	Sensor coordinate system unit
sEMG	Surface electromyography
SVM	Support vector machine
ViT	Vision transformer
